# Synthesis and Characterization of Iron Nanoparticles from a Bioflocculant Produced by *Pichia kudriavzevii* Isolated from Kombucha Tea SCOBY

**DOI:** 10.3390/bioengineering11111091

**Published:** 2024-10-30

**Authors:** Phakamani H. Tsilo, Albertus K. Basson, Zuzingcebo G. Ntombela, Nkosinathi G. Dlamini, V. S. R. Rajasekhar Pullabhotla

**Affiliations:** 1 Department of Biochemistry and Microbiology, Faculty of Science, Agriculture, and Engineering, University of Zululand, P/Bag X1001, KwaDlangezwa 3886, South Africa; 2 Department of Chemistry, Faculty of Science, Agriculture, and Engineering, University of Zululand, P/Bag X1001, KwaDlangezwa 3886, South Africa

**Keywords:** characterization, *Pichia kudraivzevii*, SCOBY, biosynthesis, iron nanoparticles, bioflocculant

## Abstract

The intriguing characteristics of nanoparticles have fueled recent advancement in the field of nanotechnology. In the current study, a microbial-based bioflocculant made from the SCOBY of Kombucha tea broth was purified, profiled, and utilized to biosynthesize iron nanoparticles as a capping and reducing agent. UV–visible absorption spectroscopy, transform infrared spectroscopy (FT-IR), X-ray diffraction (XRD), transmission electron microscopy (TEM), scanning electron microscopy (SEM), energy-dispersive X-ray analysis (EDX), and TGA were used to characterize the Fe nanoparticles. The FT-IR spectra showed functional groups such as hydroxyl, a halogen (C-Br), and carbonyl, and the alkane (C-H) functional groups were present in both samples (bioflocculant and FeNPs) with the exception of the Fe-O bond, which represented the successful biosynthesis of FeNPs. The TEM investigation revealed that the sizes of the produced iron nanoparticles were between 2.6 and 6.2 nm. The UV-vis spectra revealed peaks at 230 nm for the bioflocculant and for the as-fabricated FeNPs, peaks were around 210, 265, and 330 nm, which confirms the formation of FeNPs. X-ray diffraction presented planes (012), (104), (110), (113), (024), (116), and (533) and these planes correspond to 17.17, 32.58, 33.75, 38.18, 45.31, 57.40, and 72.4° at 2Ө. The presence of Fe nanoparticles presented with 0.82 wt% from the EDX spectrum of the biosynthesized FeNPs. However, Fe content was not present from the bioflocculant. SEM images reported cumulus-like particles of the bioflocculant, while that of FeNPs were agglomerated and hexagonal with sizes between 18 and 50 nm. The TGA of FeNPs showed thermal stability by retaining above 60% of its weight at high temperatures. It can therefore be deduced that the purified bioflocculant produced by a yeast *Pichia kudraivzevii* can be utilized to synthesize FeNPs with the current simple and effective method.

## 1. Introduction

Nanotechnology is the practice of observing, manipulating, and fabricating objects on an atomic or molecular scale, typically between one and one hundred nanometers. Nanomaterials have a huge surface area-to-volume ratio, which is the most essential attribute that explains their broad usage in optics, microbiology, electronics, medicine, environmental remediation, mechanics, and a variety of engineering fields, as well as materials science [1]. Various methods have been developed to produce metallic nanoparticles across various disciplines. The top-down and bottom-up techniques are the two approaches currently employed to manufacture nanoparticles [2]. The top-down method, which typically uses lithographic techniques and mechanical processes like grinding and milling to create nanoparticles, is more common than the bottom-up method, which uses chemical synthesis to combine small building blocks into larger structures [3]. The bottom-up strategy, in which a nanoparticle is “grown” from simpler molecules known as reaction precursors, is the most acceptable and effective method for nanoparticle synthesis. By varying precursor amounts and processing conditions, it should be feasible to adjust the shape and size of nanoparticles depending on the later application, for example, temperature, pH, etc. [4,5]. Metal and metal oxide nanoparticles are produced using a variety of physicochemical processes. This process, however, necessitates the employment of highly reactive and hazardous reducing agents like hydrazine hydrate and sodium borohydrate, which have unintended negative consequences for the environment, animals, and plant life that it supports [6].

Scientists worldwide are working to develop green chemical procedures that are simple, effective, and dependable for the synthesis of nanoparticles [7]. Numerous organisms serve as clean, environmentally safe, and long-lasting precursors to produce robust and well-functionalized nanoparticles. Actinomycetes, viruses, fungi, yeasts, bacteria, and other microorganisms are some examples [8,9]. Consequently, it is critical to investigate a more dependable and long-term reliable procedure for nanomaterial fabrication. Industries must strike a delicate balance between environmentally sound “green” procedures and their long-term viability for the prices of produced nanomaterial products be accessible to customers. Green nanotechnology-based production processes work without the use of harmful chemicals in an environmentally friendly manner and without the consumption of a lot of energy.

Recent studies have demonstrated that iron nanoparticles may be used to clean up the environment [10]. In order to remove contaminants from water and wastewater, nanoscale materials including but not limited to nano-absorbents, nanofiltration, nano-catalysts, and nano-biocides are currently used rather than withholding metal and metal oxide nanoparticles [11]. One of these metallic nanoparticles with the potential for producing environmental pollution is iron nanoparticles (FeNPs). Nanoscale zero-valent iron (nZI) is becoming more reactive at the nanoscale due to its huge surface area-to-volume-ratio [12]. The development of iron nanoparticles, such as metallic iron and iron oxide, has significantly advanced thanks to the invention of a more practical “greener” method for their generation/synthesis. Thus, in recent years, bioflocculants have been developed for the creation of iron nanoparticles [13].

Due to their distinctive flocculating properties, safety, non-toxicity, and biodegradability, which contribute to the formation of an eco-friendly environment, bioflocculants have attracted a lot of interest [14]. Bioflocculants act as a better alternative to flocculants made from chemicals [15], and they have a wide range of applications such as in the synthesis of nanomaterials, the elimination of heavy metals and dyes, the processing of minerals, the treatment of drinking water and wastewater, etc. [16,17]. Bioflocculants have been produced from microorganisms isolated from different sources such as marine sources, soil, mine water, estuaries, and Kombucha tea [18]. The current study isolated a yeast *Pichia kudriavzevii* from Kombucha tea SCOBY and produced a bioflocculant capable of synthesizing iron nanoparticles. Black tea leaves are fermented to create the sweetened beverage known as Kombucha tea [19]. Sometimes, oolong or green tea leaves are also employed. A SCOBY is a by-product of the fermentation of Kombucha, which is a biofilm made from cellulose that contains a bacterial and yeast symbiotic culture [20,21]. Numerous studies on the Kombucha SCOBY are currently being carried out to investigate all the potential applications of this cellulose as a viable raw material in industries like food technology, the development of biomaterials, the fashion and textile industries, environmental biotechnology, and many others [22]. In this current paper, the synthesis of iron nanoparticles in a very simple and safer way using a bioflocculant for capping and stabilizing is presented.

## 2. Materials and Methods

### 2.1. Isolate Activation for Fermentation

The Kombucha tea SCOBY sample was bought from GreenHeart Organics in Pine Town, South Africa. The Kombucha tea broth with the SCOBY was then serially diluted ten times in the laboratory using 0.85% saline solution. A Kombucha tea broth in an amount of 1 mL was added to 9 mL of sterilized saline solution and stirred for 30 s. Then, 100 mL of the solution was subjected to serial dilutions (1 × 10^−1^ to 10^−10^). The activation medium was prepared by mixing 10 g of tryptone, 5 g sodium chloride, and 3 g beef extract in a liter of Kombucha tea broth. Approximately, 5 mL of the activation medium was added to several test tubes before being autoclaved for 15 min at 121 °C. Following autoclaving of the activation medium, the isolate was inoculated into cooled broth tubes and incubated at 37 °C with a shaking speed of 160 revolutions per minute (rpm) for 60 h. There were about ten isolates screened for potential bioflocculant production. The isolate that showed a high flocculation rate was identified and showed similarities of 99% to yeast strain *Pichia kudriavzevii*, and this isolate was chosen as the organism of choice. The bioflocculant produced from the isolate was 2.836 g/L, which is comparable to other reports from the literature.

### 2.2. Extraction and Purification of the Bioflocculant

The pure bioflocculant was extracted as outlined in the method by Ngema et al. [23]. Following fermentation of the culture medium for 72 h at 35 °C, the mixture was centrifuged at 8000 rpm for 15 min at 4 °C. The supernatant was then collected, mixed with 2 L of ice-cold ethanol for extraction, and left to precipitate for 12 h at a temperature of 4 °C. The resultant dried crude bioflocculant was dissolved in 100 mL of deionized water; thereafter, a mixture of chloroform and butanol (5:2 *v*/*v*) was added and allowed to sediment for 12 h before the precipitate was collected and dried in a vacuum to obtain a pure bioflocculant.

### 2.3. Biosynthesis of Iron Nanoparticles (FeNPs)

For the fabrication of iron nanomaterials, iron sulfate (FeSO_4_) was utilized as the metal precursor. Following the procedure outlined by Dlamini et al. (2020b), 0.5 g of purified bioflocculant was dissolved in a 0.2 M FeSO_4_ solution, and 10 mL of a 0.5 M NaOH solution was added to prevent nanomaterial aggregation to generate the FeNPs. When the combination was stirred and left at room temperature overnight, a color shift from colorless to a brownish color was noticed, which indicated that nanoparticles had formed. The mixture underwent a 15 min centrifugation process at 5000 rpm and at a temperature of 4 °C to separate the biosynthesized nanoparticles from the solution. The subsequent precipitate was vacuum-dried for 24 h at room temperature. To verify the biosynthesis of iron nanoparticles, the powder was physically observed and characterized. The Fourier transform infrared (FT-IR) spectroscopy, ultraviolet–visible (UV-Vis) spectroscopy, X-ray diffraction (XRD), transmission electron microscopy (TEM), scanning electron microscopy (SEM), energy-dispersive X-ray (EDX) analysis, and thermogravimetric analysis (TGA) methods were used for characterization in this study. Briefly, a bioflocculant in this study is a product extracted from an isolate, *Pichia kudriavzevii*, and this organism was able to produce this product after it was cultivated in nutrient-rich media. On the other hand, iron nanoparticles (FeNPs) were synthesized from a bioflocculant using the “green” synthesis route. The product (FeNPs) gained properties of the bioflocculant used as a stabilizer or reducing agent and the properties of metal iron.

### 2.4. Characterization of Biosynthesized FeNPs

To confirm the functional groups present in the iron nanoparticles produced, FT-IR Tensor 27 spectroscopy (Bruker, Johannesburg, South Africa) was performed. The iron nanoparticles were analyzed using a potassium bromide pelleting process at a 1:100 ratio through FT-IR and ranged from 400 to 4000 cm^−1^.

To ascertain the nature and average size of the generated iron nanoparticles from the aqueous bioflocculant, an X-ray diffraction (Brucker AXS D8 Advance diffraction, Johannesburg, South Africa) examination was performed. The powdered samples were placed on a flat sample holder, and the scan range was from 5 to 90 2θ, with steps of 0.05 in a 0.03 s scan rate and a 0.00657 s step size.

The nanoparticles were examined and their surface shapes and morphologies were determined using scanning electron microscopy (SEM) (JOEL USA, Inc., Peabody, MA, USA).

The as-synthesized iron nanoparticles were further examined by energy-dispersive X-ray (EDX) analysis (SEM) (JOEL USA, Inc., Peabody, MA, USA) to ascertain the elemental composition after drying on a copper grid coated with carbon.

The biosynthesized iron nanoparticles were submitted to the thermogravimetric analysis using a PerkinElmer Thermal Analysis Pyris 6 TGA (PerkinElmer, Inc., Waltham, MA, USA). The temperature ranges from 22 to 900 °C was used at a rate of 10 °C/min, while the nitrogen gas flow rate was kept constant at 40 cc/min.

A 1 mL aliquot of colloidal iron nanoparticle solution in quartz cuvettes was evaluated using UV–visible spectroscopy with de-ionized water as a reference and 0.05 mM FeSO_4_ as a blank with a range from 200 to 700 nm to confirm the reduction of the ferric ions in the colloidal solution.

## 3. Results and Discussions

The traditional protocol for the fabrication of nanoparticles, including sol–gel, hydrothermal, and sonochemical methods, shows several drawbacks, including an increased cost of manufacturing, low rates of production, and significant energy requirements (Ref). Chemical methods (such as sonochemical, hydrothermal, precipitation, etc.) involve the utilization of hazardous chemicals, the production of dangerous by-products, and contamination from hazardous materials. Therefore, it is necessary to generate nanoparticles that are pure, have no toxicity effects, and are environmentally friendly and utilize biomass waste from plants and microorganisms as reductants. The iron nanoparticles (FeNPs) synthesized in the present study showed no toxicity effects and the method of synthesis was cheap, though much time was invested for microbial growth.

### 3.1. The FT-IR Analysis

**Figure 1:**
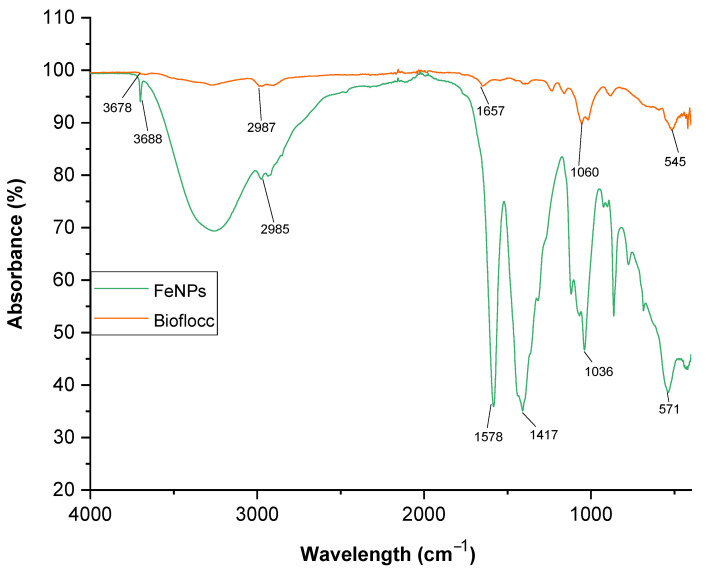
FT-IR spectra of the bioflocculant and the as-synthesized iron nanoparticles.

Figure 1 displays FT-IR spectra for the bioflocculant and biosynthesized iron nanoparticles. The hydroxyl (-OH) group is contained in the samples, as indicated by the band at 3678 cm^−1^ in the bioflocculant, and this band is also observed in the FeNPs. The alkane group is characterized by a C-H bond at the band value of 2987 cm^−1^. The stretching of C-O bonds is shown by the two bands in the regions 1657 cm^−1^ and 1060 cm^−1^. It has been reported that these functional groups play a role in the synthesis of iron nanoparticles as capping and reducing agents [24]. These bands are indicative of the polysaccharides present in the bioflocculant. It is claimed that biological substances like bioflocculants have functional groups that can reduce metal salts to generate nanoparticles [25]. Additionally, functional groups serve as the sites where suspended particles can bind to form flocs during flocculation [26]. The FT-IR spectra of FeNPs revealed a stretching vibration at 3688 cm^−1^, indicating the presence of a -OH functional group. Another absorption peak was observed in a FeNP molecule at a band value of 2985 cm^−1^ for the stretching vibration of phenol groups’ -OH, which might be assigned for the stabilization and formation of FeNPs. The FT-IR spectrum of the FeNPs showed a C=O stretching vibration at 1578 cm^−1^, which could be attributed to the carbonyl group presence. Some bands were observed at 1417 cm^−1^ and 571 cm^−1^, which indicate the C-O-C and halogen compound (C-Br), respectively. These functional groups served as the site for the solution’s positively charged metal ions to bind to, leading to the formation of FeNPs. Üstün et al. [27] also reported an absorption peak at 576 cm^−1^, confirming the FeNP synthesis. The findings of this study agree with those reported by Katata-Seru et al. [28], where iron nanoparticles were synthesized using *Moringa oleifera*. Their FT-IR results showed peaks at band values of 3519, 2918, 1615, 1408, 1049, and 567 cm^−1^. Tyagi et al. [29] also reported similar functional groups with the bands at 3418, 3318, 2508, 1438, and 540 cm^−1^ for FT-IR spectra of FeNPs synthesized using banana and spinach leaf extracts. The mechanism by which the purified bioflocculant reduces ferric ions works by the donation of electrons contained in the functional groups, including carboxyl and hydroxyl groups; this converts Fe^3+^ ions to Fe^2+^, which eventually leads to Fe^o^. This has been likened to antioxidant activity as electron transfer occurs through the redox process. Through surface capping, the bioflocculant stabilizes the resulting nanoparticles.

### 3.2. UV–Visible Spectroscopy Analysis

**Figure 2:**
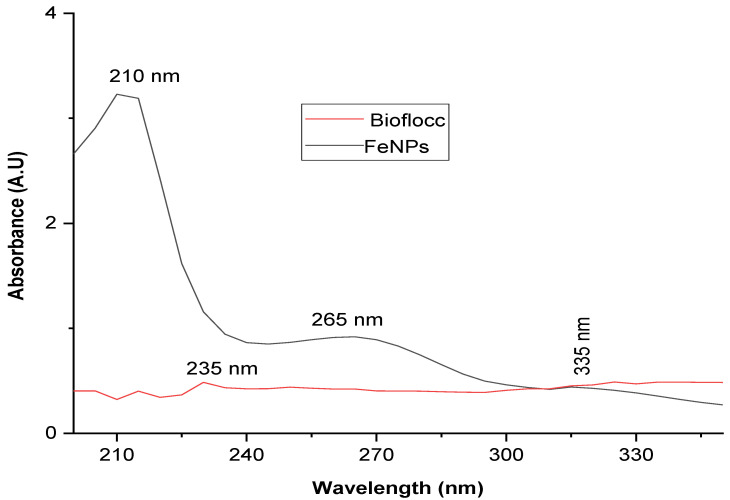
UV–visible spectra of bioflocculant and as-synthesized iron nanoparticles.

The UV-vis spectra of the bioflocculant and the biosynthesized FeNPs are shown in Figure 2. The bioflocculant showed an absorption peak at 235 nm (Figure 2). When FeSO_4_ and the bioflocculant reacted, the color of the reaction mixture quickly changed from yellow to a dark color. The suspension of the precursor solution’s color changed from yellow to a dark color, indicating that Fe^2^ and Fe^3^ ions had undergone bioreduction [30]. This is attributed to the synthesis of FeNPs that result from the polyphenols present in the bioflocculant-reducing trivalent iron [31]. In a study by Ting and Chin [32], it was reported that the precursor solutions (pH 1.8) generated a greenish-brown suspension (pH 2.3) after the addition of apple peel extract (APE) (bioflocculant), and this suspension changed into a greenish-black suspension (pH 12.0) after the addition of 20 mL of NaOH. The bioactive compounds present in plant extract from a study by Roy et al. [33] resulted in a change in color of the FeSO_4_ from bluish-green to yellow after the addition of the plant extract. This might be because of a surface plasmon resonance shift that shows the conversion of bulk iron into iron nanoparticles. The strength of the peaks at 210 and 265 nm decreased after reacting with Fe^2+^ when the reaction between FeSO_4_ and the bioflocculant led to the color change from yellow to a brownish color, confirming biosynthesis [34]. Another small hump at 335 nm was revealed from the biosynthesized FeNPs. A possible explanation for the absorbance at around 335 nm is the presence of impurities or contaminants in the sample. This suggests that the iron nanomaterials generated by the bioflocculant were capped and reduced during the synthesis [35,36]. Furthermore, the tiny FeNP clusters can be attributed to this hump (335 nm), which is consistent with the absorption spectra reported by Alqudami and Annapoorni [37]. Saini et al. [38] reported the absorbance peak of iron nanoparticles in the 210–260 nm range. The UV–visible spectroscopy of *urtica dioica*-derived biosynthesized iron nanoparticles showed absorption in the 216–265 nm range [39].

### 3.3. X-Ray Diffraction Analysis

**Figure 3:**
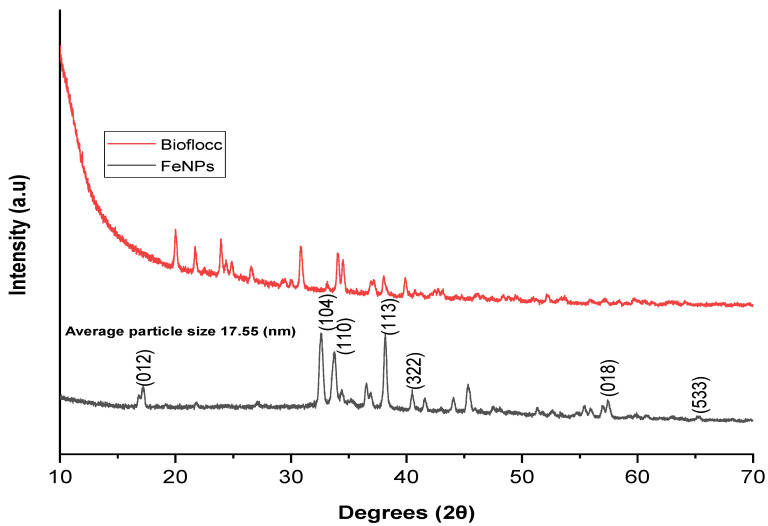
Investigation of iron nanoparticles and bioflocculant by X-ray diffraction.

XRD spectroscopy is a potent method for displaying the crystalline and amorphous domains in the particles. A powder X-ray diffraction analysis was used to characterize the materials’ structural properties. Figure 3 indicates the diffraction patterns for both the bioflocculant and the as-synthesized FeNPs. The bioflocculant showed small diffraction peaks which were not broad at 2θ = 20°, 23°, 25°, 30°, and 34°. This signifies that the bioflocculant has bigger particles compared to iron nanoparticles [40].

A JCPDS CARD No. 089-2810, which corresponds to Fe_2_O_3_ in rhombohedral geometry, was revealed to be in good agreement with all the diffraction peaks in this diffraction for the as-synthesized FeNPs [41]. At peak values of 17.17°, 32.58°, 33.75°, 38.18°, 45.31°, 57.40°, and 72.4°, the sample displayed the characteristic peaks for produced crystalline FeNPs. The planes (012), (104), (110), (113), (024), (116), and (533), respectively, are associated with these peaks. The peak observed at 45.31° 2θ is associated with the trace amount of β-Fe_2_O_3_ present in the particles, which further indicates that the iron nanoparticles are crystalline in nature [42]. Balamurugan et al.’s [43] observations were similar, where *Eucalyptus globulus* was utilized to biosynthesize iron oxide nanoparticles. Anchan et al. [44] also documented similar results from the XRD spectra of CP-FONPs. The bioflocculant’s polyphenols are represented by a modest peak at 17.17° in the FeNP pattern [45]. Jagathesan et al. [46] reported the XRD of FeNPs with diffraction peaks at 2θ = 30°, 37°, 42°, 54°, 58°, and 63°, which corresponded to the crystal planes of (220), (311), (400), (422), (511), and (440), respectively, which is contrary to this study findings. The presence of other peaks from the sample indicated that the particles are highly pure [44]. Using Scherrer’s equation, the mean crystallite size of the as-synthesized FeNPs was found to be 17.55 nm. An average particle size of 29 nm was reported by Khalil et al. [47] for the synthesis of Fe_2_O_3_ utilizing *Sageretia thea* extracts.

### 3.4. Thermogravimetric Analysis

**Figure 4:**
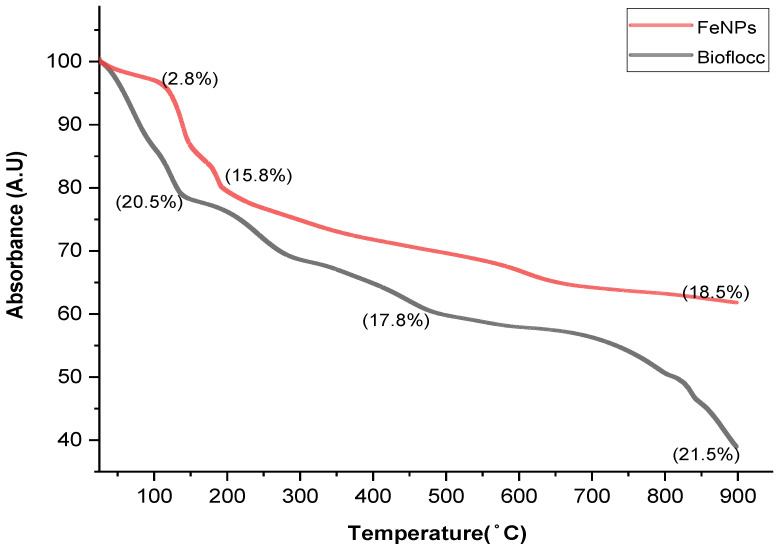
Thermogravimetric analysis of bioflocculant and as-synthesized iron nanoparticles. (%) on the graph represents weight percentage loss.

Figure 4 shows the TG pattern for both the bioflocculant and FeNPs. The thermogravimetric pattern shows a three-phase disintegration pattern for both samples. During the first stage for the biosynthesized FeNPs, weight loss (2.8%wt) occurred at about 50–100 °C, while that of the bioflocculant (20.5%wt) was observed around 50–150 °C. This mass loss could be because of the drying-out of the solvents (from the surface of the particles) utilized during purification. The presence of the second stage between 150 and 210 °C (FeNPs) and 150–500 °C (bioflocculant) may be assigned to the polymer’s breakdown and the removal of organic residues from the samples, which led to a weight loss of 15.8% for the as-prepared FeNPs and 17.8% for the bioflocculant. The third stage is observed at about 200–900 °C for the FeNPs and from 500 to 900 °C for the bioflocculant. A further degradation of about 18.5% was observed for the as-prepared FeNPs, and the bioflocculant lost about 21.5% more of its mass. At about 900 °C, the as-prepared FeNPs retained above 60% of its mass, thus suggesting that it is thermally stable, while the bioflocculant only retained about 40% of its mass. The reason behind FeNPs having more mass retained over the bioflocculant could be because of the properties of both the bioflocculant and the iron that have been imparted to the formation of a thermally stable molecule (FeNPs). Additionally, it has been reported that smaller and more stable nanoparticles form because of the high temperatures [48]. Hema et al. [49] reported TGA spectra for iron nanoparticles, which revealed that at temperatures above 700 °C, the FeNPs retained about 67% of its mass, indicating its stability at high temperatures.

### 3.5. TEM Analysis

**Figure 5:**
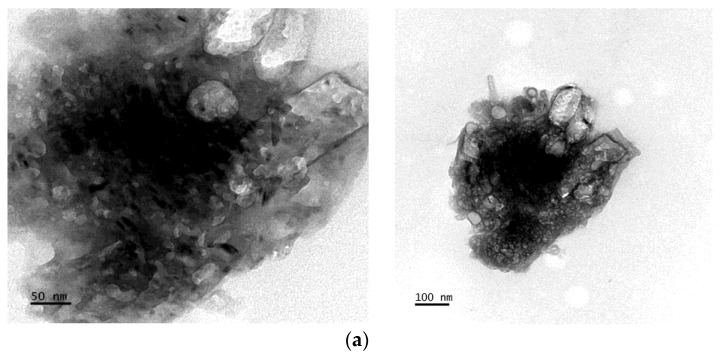

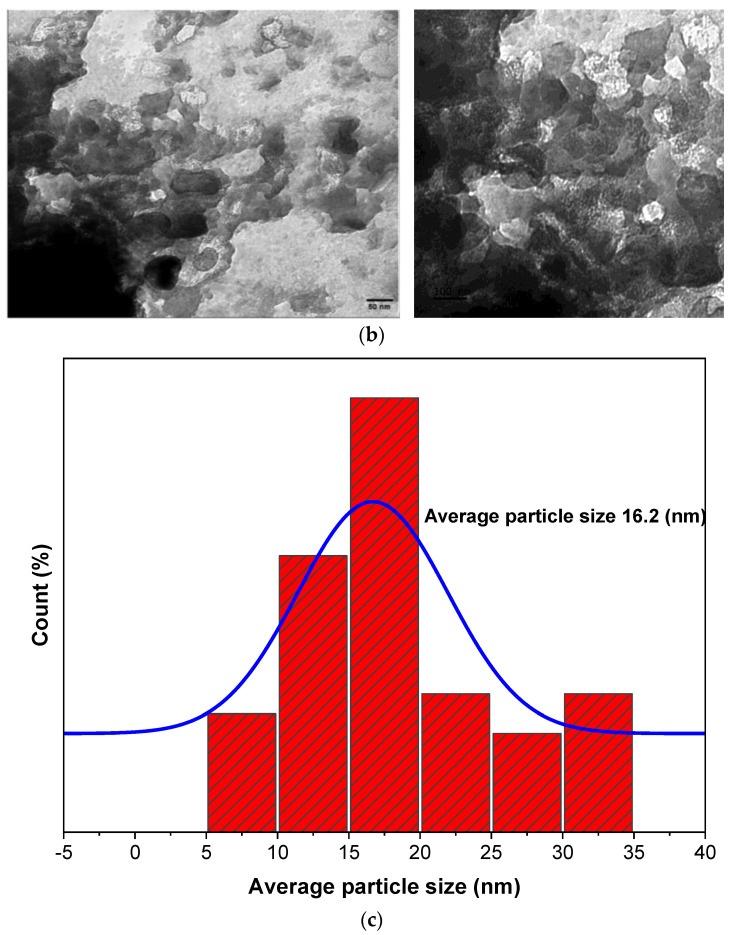
TEM images of (**a**) the bioflocculant and (**b**) as-prepared iron nanoparticles. (**c**) Histogram for the biosynthesized FeNPs.

Using transmission electron microscopy, the size and shape of the bioflocculant and the as-prepared FeNPs were examined, and their images are depicted in Figure 5. The TEM image in Figure 5a reveals that the iron nanoparticles have a thick surface layer and are close to spherical. The size variation in the FeNPs was determined to be between 16.2 and 18.55 nm by a transmission electron microscope. Yadev et al. [50] documented a similar set of results by defining the spherical shape of Aloe vera-derived FeNPs. The bioflocculant (Figure 5b) revealed particles that are close to spherical, with an average particle size between 17.44 and 19.5 nm, which could be the result of the substrate bioflocculant concentration and substrate properties [51]. Xu et al. [51], through a TEM micrograph, reported a bioflocculant with various morphologies, including thin films, aggregates with string-like nanofibers, and nanotubes. Figure 5c shows the histogram of the synthesized FeNPs; these particles are predominantly sized around 16.2 nm, with a centered normal distribution around this value. Most of the nanoparticles range between 10 and 20 nm in size.

### 3.6. SEM Analysis

**Figure 6:**
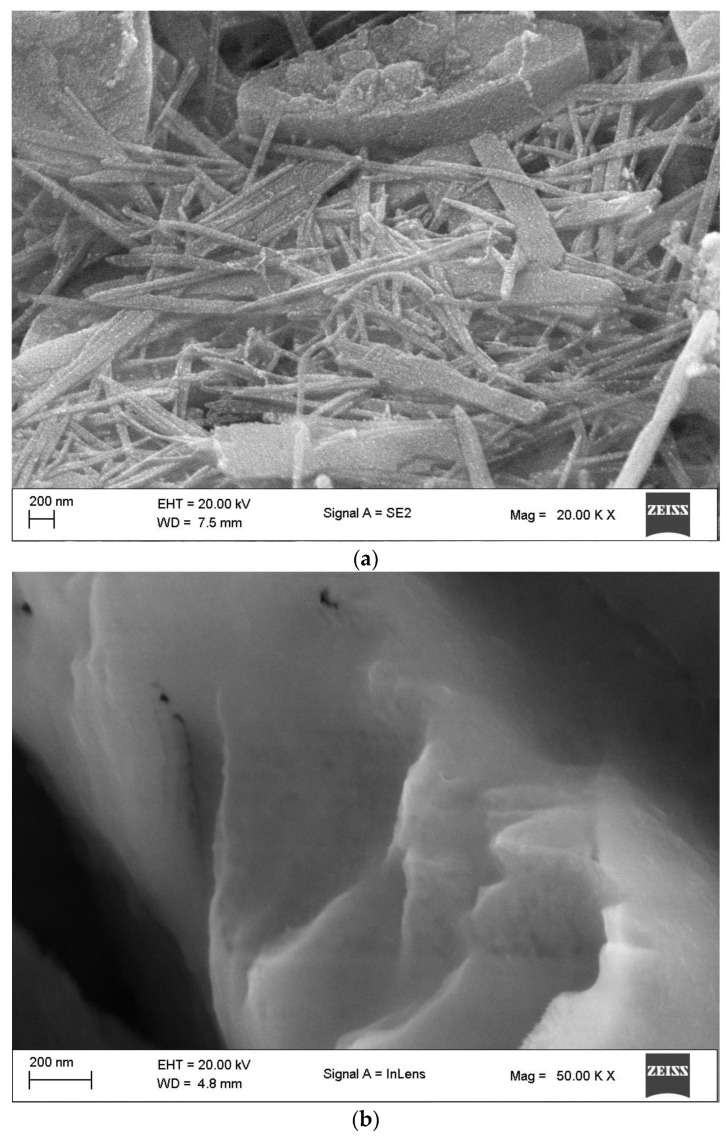
SEM images of (**a**) as-synthesized iron nanoparticles and (**b**) the bioflocculant.

Iron nanoparticles generated with a bioflocculant produced from *Pichia kudriavzevii* and the bioflocculant were examined using scanning electron microscopy, as depicted in Figure 6. The bioflocculant showed an irregular fibrous rod-like shape, which indicates the presence of elongated or needle-like particles with an average particle size of 14 nm to 53 nm (Figure 6b). Conversely, the morphology of the biosynthesized FeNPs comprises smooth-layered structures, and some irregularity is also observed with some possible folding. This could be due to the appearance of the bioflocculant used for synthesis (Figure 6a). These structures could be aggregated nanoparticles or crystalline arrangements, which are common characteristics of nanoparticles produced through biological synthesis [52]. The particle size of the FeNPs was between 18 and 53 nm. A similar report was documented when a bioflocculant from *Lawsonia inermis* and *Gardenia jasminoides* leaf extract was utilized to synthesize FeNPs [53], and their SEM image revealed particles that were agglomerated due to the adhesive nature of the plant extract; the morphology was a distorted hexagonal-like appearance having particles sizes between 21 and 32 nm. In another study, spherical-shaped particles were observed for iron nanoparticles with an average size of 45–100 nm [54]. Particles with a round shape and with a mean size of 20 nm, as revealed by scanning electron microscopy, were reported by Xu et al. [55].

### 3.7. EDX Analysis

**Figure 7:**
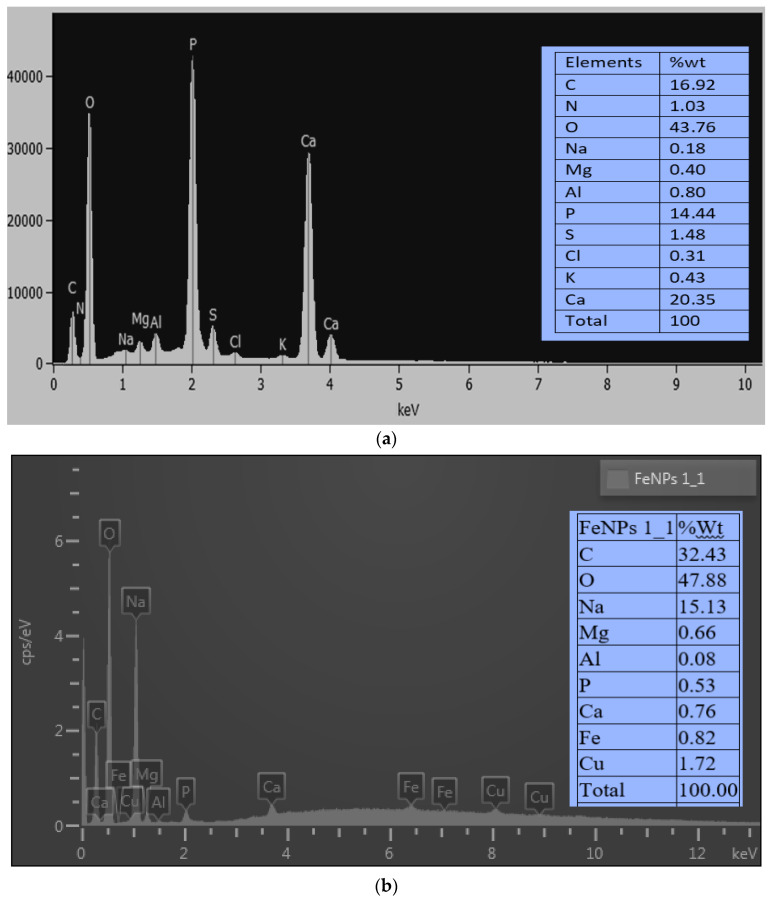
EDX analysis of (**a**) the bioflocculant and (**b**) as-synthesized iron nanoparticles.

Energy-dispersive X-ray analysis was used to examine the chemical make-up of the bioflocculant and the biosynthesized iron nanoparticles. The bioflocculant from *Pichia kudravzevii* showed an EDX spectrum with the presence of carbonaceous matter, having carbon with 16.92%wt, nitrogenous material with 1.03%wt, some oxygen with 43%wt, and other elements (Figure 7a). The presence of C and O in the bioflocculant signifies that the compound is a carbohydrate polymer. Corresponding results were documented by Shende and Mitra [14], where they utilized an Okra bioflocculant to synthesize Fe nanoparticles. They reported elements such as C and O, with 51 and 42.66%wt, respectively, among others present. Figure 7b displays an EDX spectrum of the biosynthesized FeNPs. The biosynthesized Fe nanoparticles contain elements including C, O, and Fe, as indicated by the EDX analysis, and their corresponding percentage weights were 32.43, 47.88, and 0.82%wt, respectively. The presence of iron in the FeNPs EDX spectra confirms the successful biosynthesis of FeNPs, as this element is not shown in the bioflocculant spectrum (Figure 7a). Jagathesan and Rajiv [46] reported that the biosynthesized FeNPs contained an iron content and oxygen content of 77.08%wt and 22.97%wt, respectively. Roman nettle-mediated FeNPs revealed to be composed of 61.2% iron, 17.2% oxygen, and 21% other components [56].

## 4. Conclusions

A purified bioflocculant from *Pichia kudriavzevii* was utilized to successfully biosynthesize iron nanoparticles, and they were then characterized using different methods such as FT-IR, UV–visible spectroscopy, TEM, SEM, and EDX analysis. The FT-IR analysis revealed functional groups for both the bioflocculant and the biosynthesized FeNPs. The FT-IR spectra revealed the presence of functional groups such as hydroxyl, carboxyl, carbonyl, alkanes, and Fe-O. The UV-vis analysis showed peak maxima at 235 nm for the bioflocculant and for the biosynthesized iron nanoparticles, peaks were observed at 210, 265, and 335 nm, confirming the successful formation of FeNPs from the bioflocculant. TEM showed that the biosynthesized FeNPs were spherically formed and had a thick surface layer with a particle size ranging from 16.2 to 18.55 nm. Thermogravimetric analysis demonstrated that the as-prepared FeNPs are thermally stable, as they retained 60% of their mass at high temperatures. SEM images showed the hexagonal shape of the particles, while EDX confirmed the presence of Fe, which was not present in the bioflocculant spectra; other elements present were C, O, and Na in the FeNPs. The XRD pattern showed characteristic peaks that correspond to the planes (012), (104), (110), (113), (024), (116), and (533), and the particle size was 17.55 nm.

## Data Availability

No new data was generated in the study.
